# Ligand-receptor dynamics in heterophily-aware graph neural networks for enhanced cell type prediction from single-cell RNA-seq data

**DOI:** 10.3389/fmolb.2025.1547231

**Published:** 2025-05-12

**Authors:** Lian Duan, Mahshad Hashemi, Alioune Ngom, Luis Rueda

**Affiliations:** School of Computer Science, University of Windsor, Windsor, ON, Canada

**Keywords:** graph neural networks, single-cell RNA sequencing, cell-cell communication, heterophily, homophily, cell type prediction

## Abstract

Graph Neural Networks (GNNs) have emerged as powerful tools for analyzing structured data, particularly in domains where relationships and interactions between entities are key. By leveraging the inherent graph structure in datasets, GNNs excel in capturing complex dependencies and patterns that traditional neural networks might miss. This advantage is especially pronounced in the field of computational biology, where the intricate connections between biological entities play a crucial role. In this context, Our work explores the application of GNNs to single-cell RNA sequencing (scRNA-seq) data, a domain characterized by complex and heterogeneous relationships. By extracting ligand-receptor (L-R) associations from LIANA and constructing Cell-Cell association networks with varying edge homophily ratios, based on L-R information, we enhance the biological relevance and accuracy of depicting cellular communication pathways. While standard GNN models like Graph Convolutional Networks (GCN), GraphSAGE, Graph Attention Networks (GAT), and MixHop often assume homophily (similar nodes are more likely to be connected), this assumption does not always hold in biological networks. To address this, we explore advanced graph neural network methods, such as ${\text{H}}_{2}$ Graph Convolutional Networks and Gated Bi-Kernel GNNs (GBK-GNN), that are specifically designed to handle heterophilic data. Our study spans across six diverse datasets, enabling a thorough comparison between heterophily-aware GNNs and traditional homophily-assuming models, including Multi-Layer Perceptrons, which disregards graph structure entirely. Our findings highlight the importance of considering data-specific characteristics in GNN applications, demonstrating that heterophily-focused methods can effectively decipher the complex patterns within scRNA-seq data. By integrating multi-omics data, including gene expression profiles and L-R interactions, we pave the way for more accurate and insightful analyses in computational biology, offering a more comprehensive understanding of cellular environments and interactions.

## 1 Introduction

In the rapidly evolving field of computational biology, the integration of Graph Neural Networks (GNNs) with single-cell RNA sequencing (scRNA-seq) data heralds a significant leap forward. scRNA-seq, known for its unparalleled ability to illuminate cellular distinctions and tissue compositions, faces considerable computational hurdles due to its intrinsic complexity and heterogeneity. Traditional GNN architectures like Graph Convolutional Networks (GCN) introduced by Kipf and Welling (Kipf and Welling, 2016), Graph Attention Networks (GAT) (Veličković et al., 2018), GraphSAGE developed by Hamilton et al. (Ying et al., 2017), and MixHop, introduced by Abu-El-Haija et al. (Abu-El-Haija et al., 2019), have demonstrated their utility across various domains by harnessing network structural information and individual node characteristics. However, these models predominantly assume homophily—the tendency for similar nodes to be more closely connected—an assumption that does not always hold in the diverse and intricate biological networks characterized by both homophilic and heterophilic relationships. The foundational GCN model, as proposed by (Kipf and Welling, 2016), excels under the homophily assumption, where like nodes are more likely to be connected. Yet, this premise may falter in the realm of biological networks, where interactions frequently occur between dissimilar entities, underscoring the necessity for models adept at managing heterophily. Addressing this gap, Hamilton et al. (Ying et al., 2017) extended GCNs with GraphSAGE, introducing an inductive learning framework that enhances adaptability to dynamic or evolving networks, a critical feature for biological systems where new cellular states or types continually emerge. Recent advancements have led to the development of more specialized GNNs to navigate the nuanced landscape of biological data. ${\text{H}}_{2}$ GCN, crafted by Zhu et al. (Zhu et al., 2020) specifically for heterophilic data, employs diverse neighborhood aggregation strategies to effectively capture the complex interplay of interactions typical in biological datasets. In a similar vein, the Gated Bi-Kernel GNN (GBK-GNN), conceived by Du et al. (Du et al., 2022), pioneers a dual-kernel approach that meticulously processes both homophilic and heterophilic relationships, significantly augmenting the model’s interpretive power in deciphering complex biological networks. Contrasting with these graph-based approaches, Multi-Layer Perceptrons (MLP), which process node features in isolation from the graph structure, serve as a fundamental baseline, highlighting the intrinsic value of relational information in graph-based learning paradigms.

This study systematically applies and juxtaposes these models across a spectrum of scRNA-seq datasets, utilizing biologically informed interaction graphs constructed with LIANA (LIgand-receptor ANalysis frAmework) (Türei et al., 2022). LIANA (Türei et al., 2022) provides a framework for inferring Cell-Cell Communication (CCC) pathways by identifying ligand-receptor (L-R) interactions based on scRNA-seq data. By integrating multiple established methods, LIANA (Türei et al., 2022) generates a cell-cell adjacency graph where nodes represent individual cells, and edges signify potential communication pathways based on L-R signaling. This approach allows us to construct interaction networks that more accurately reflect the underlying biology, setting the stage for a meaningful application of advanced GNNs. Through this endeavor, we aspire to illuminate the strengths and limitations inherent in each model within the context of computational biology, thereby furnishing valuable insights into their practical applicability and steering future research directions in the field. This exhaustive analysis stands to make a substantial contribution to our understanding of cellular functionalities and interactions, leveraging the sophisticated methodologies of advanced GNNs alongside interaction networks inferred through LIANA (Türei et al., 2022).

### 1.1 Homophily and heterophily

**Homophily** and **heterophily** are concepts in network theory and graph analysis that describe the trends of edges in a graph to connect nodes with similar or dissimilar attributes, respectively. Formally, consider an undirected graph G=(V,E) , where V represents the set of nodes and E⊆V×V denotes the set of edges. Each node v∈V is associated with a feature vector $x_{v}\in R^{d}$ or a categorical label $y_{v}\in Y$ , where Y is the set of possible classes or categories.

**Edge homophily** quantifies the propensity of edges to connect nodes that share similar attributes or labels. Mathematically, homophily can be measured using the *homophily ratio* H , defined as:

$$H=\frac{\left|\left\{\left(u,v\right)\in E|y_{u}=y_{v}\right\}\right|}{\left|E\right|}$$ (1)

In Equation 1, H represents the fraction of edges that link nodes with identical labels. A homophily ratio H>0.5 indicates a strong tendency for nodes to connect with similar nodes, reflecting assortative mixing within the graph.

**FIGURE 1:**
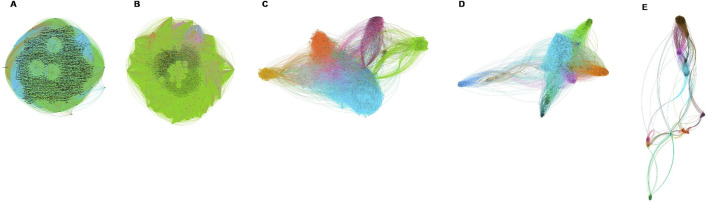
Visualization of the constructed graphs with five H ratio levels **(A)** 0–0.2, **(B)** 0.2–0.4, **(C)** 0.4–0.6, **(D)** 0.6–0.8, **(E)** 0.8–1. At the highest homophily level **(E)**, the visualization clearly shows sparse clusters of cells, where each cell predominantly connects with others of the same type (same colors), illustrating strong homophily. As the H ratio decreases towards **(A)**, the distinction between clusters becomes increasingly blurred, leading to a more mixed and less defined structure in the graphs.

Both homophily and heterophily are intrinsic properties of the edges in a graph, as they directly relate to the nature of connections between node pairs. These concepts are critical in various graph-based machine learning tasks, such as node classification, link prediction, and community detection. We visualized graphs to illustrate homophily using Gephi (Bastian and Gephi, 2009) shown in Figure 1. In the context of GNN, the degree of homophily or heterophily influences the effectiveness of message-passing mechanisms. For instance, in heterophilic graphs, aggregating features from diverse neighbors may necessitate more sophisticated aggregation functions or attention mechanisms to capture the dissimilarity effectively.

### 1.2 Heterophily-aware methods

**Heterophily-aware methods** are specialized algorithms within the domain of graph-based machine learning that are explicitly designed to effectively handle graphs exhibiting heterophily. Unlike traditional GNNs that aggregate information primarily from neighboring nodes N(v) , heterophily-aware methods often incorporate mechanisms such as adaptive aggregation, feature transformation, or dual-pathway processing to mitigate the negative impact of aggregating dissimilar node features.

Several advanced heterophily-aware methods have been proposed to enhance GNN performance on heterophilic graphs.

#### 1.2.1 H_2_GCN

$H_{2}$ GCN (Zhu et al., 2020) is designed to perform effectively in both homophily and heterophily settings by incorporating three key designs.

**Ego- and Neighbor-Embedding Separation**: This design keeps a node’s embedding distinct from its neighbors’ embeddings, avoiding oversmoothing in heterophily. Formally, for each node v at layer k :

$$r_{v}^{\left(k\right)}=\text{COMBINE}\left(r_{v}^{\left(k-1\right)},\text{AGGR}\left(\left\{r_{u}^{\left(k-1\right)}:u\in N\left(v\right)\right\}\right)\right),$$ (2)

where AGGR is the aggregation function for neighbor embeddings, and COMBINE merges ego and neighbor representations.

**Higher-Order Neighborhoods**: To capture both local and global graph structures, ${\text{H}}_{2}$ GCN aggregates information from i -hop neighborhoods:

$$\begin{array}{ll} r_{v}^{\left(k\right)}=\text{COMBINE} & \left(\text{AGGR}\left(\left\{r_{u}^{\left(k-1\right)}:u\in N_{1}\left(v\right)\right\}\right),\right.\\ & \ldots ,\\ & \left.\text{AGGR}\left(\left\{r_{u}^{\left(k-1\right)}:u\in N_{i}\left(v\right)\right\}\right)\right), \end{array}$$ (3)

where $N_{i}\left(v\right)$ represents the i -hop neighbors of node v .

**Combination of Intermediate Representations**: ${\text{H}}_{2}$ GCN combines outputs from multiple layers to capture information at different scales:

$$r_{v}^{\text{final}}=\text{COMBINE}\left(r_{v}^{\left(0\right)},r_{v}^{\left(1\right)},\ldots ,r_{v}^{\left(K\right)}\right),$$ (4)

where $r_{v}^{\left(K\right)}$ is the representation of node embedding after K layers, and COMBINE concatenates all intermediate representations.

Finally, the classification stage uses the final embedding for prediction:

$${\hat{y}}_{v}=\text{softmax}\left(r_{v}^{\text{final}}W_{c}\right),$$ (5)

where $W_{c}$ is a learnable weight matrix. These designs enable ${\text{H}}_{2}$ GCN to adapt effectively to varying levels of homophily and heterophily by leveraging multi-scale graph information. Equations 2–5 illustrate how H2GCN (Zhu et al., 2020) balances separation, multi-hop aggregation, and multi-scale fusion to adapt to varying homophily and heterophily.

#### 1.2.2 GBK-GNN

GBK-GNN (Du et al., 2022) introduces a sophisticated mechanism that combines gating and bi-kernel approaches to effectively model both homophilic and heterophilic relationships within a graph. The model incorporates two main innovations: bi-kernel feature transformation and a kernel selection gate mechanism.

The bi-kernel transformation uses two distinct kernels: one for modeling homophily $\left(W_{s}\right)$ and the other for heterophily $\left(W_{d}\right)$ . During message passing, the kernels are combined based on a selection gate, which dynamically determines the contribution of each kernel for a node pair. Mathematically, the hidden representation $z_{i}^{\left(l\right)}$ of node i at layer l is computed as:

$$\begin{array}{ll} z_{i}^{\left(l\right)}=\sigma & \left(W_{f}z_{i}^{\left(l-1\right)}+\frac{1}{\left|N\left(v_{i}\right)\right|}\underset{v_{j}\in N\left(v_{i}\right)}{\sum }\left[\alpha_{ij}W_{s}z_{j}^{\left(l-1\right)}\right.\right.\\ & \left.\;+\left(1-\alpha_{ij}\right)W_{d}z_{j}^{\left(l-1\right)}\right]\left.\right), \end{array}$$ (6)

where $W_{f}$ , $W_{s}$ , and $W_{d}$ are learnable parameters, $\alpha_{ij}$ is the gating signal computed as:

$$\alpha_{ij}=\text{sigmoid}\left(G_{l}\left(z_{i}^{\left(l-1\right)},z_{j}^{\left(l-1\right)};W_{g}\right)\right),$$ (7)

and $G_{l}\left(\cdot \right)$ is a learnable function (e.g., an MLP). The gating mechanism helps identify whether a node pair exhibits homophily or heterophily and adjusts the kernel contributions accordingly.

The overall loss function combines the standard classification loss $\left(L_{o}\right)$ with an additional gate supervision loss $\left(L_{g}\right)$ :

$$L=L_{o}+\lambda \underset{l}{\sum }L_{g}^{\left(l\right)},$$ (8)

where λ is a hyperparameter. Equations 6–8 together enable GBK-GNN to adaptively tune its message-passing behavior across regions of varying homophily.

## 2 Materials and methods

### 2.1 Datasets

The scRNA-seq data used in this study are publicly available on Gene Expression Omnibus (GEO) with accession number of GSE84133. This dataset includes six subsets of pancreatic islets sampled from four human donors and two mice strains. The sequencing method invoked in the dataset is inDrop, a droplet-based scRNA-seq that is capable of determining the transcriptomes of over 12,000 individual pancreatic cells (Baron et al., 2016). Table 1 shows the details of dataset such as the accession number, the type of tissue sample, the number of cells, the number of genes, and the number of assigned cell types for analysis. Each dataset underwent a rigorous preprocessing pipeline, including gene filtering, normalization, and feature selection. Specifically, we retained the top 2,000 highly variable genes (HVGs) from the original count matrices to capture the most biologically informative features while reducing noise and computational complexity. The selection of 2,000 HVGs ensures that downstream models receive input features that reflect key transcriptomic variations across cells while avoiding redundancy from low-expression genes. Additionally, preprocessing steps such as removing genes expressed in fewer than 20 cells and normalizing count distributions were applied to maintain data integrity. These decisions were guided by best practices in single-cell analysis to maximize signal while ensuring computational feasibility.

**TABLE 1:** Statistics for the datasets used in this work.

Dataset	# Accession	# Tissue sample	# Cells	# Genes	# Cell types
Baron-human1	GSM2230757	Human Pancreatic Islets	1,937	20,125	14
Baron-human2	GSM2230758	Human Pancreatic Islets	1,724	20,125	14
Baron-human3	GSM2230759	Human Pancreatic Islets	3,605	20,125	14
Baron-human4	GSM2230760	Human Pancreatic Islets	1,303	20,125	14
Baron-mouse1	GSM2230761	Mouse Pancreatic Islets	822	14,878	13
Baron-mouse2	GSM2230762	Mouse Pancreatic Islets	1,064	14,878	13

### 2.2 Proposed method

Given a high-dimensional count matrix from the scRNA-seq dataset, we aim to predict cell types and compare the prediction performance of GNNs when input graphs have various levels of edge homophily ratio. The homophily ratio ( H ratio) is defined as shown in Equation 9:

$$h_{\mathrm{ratio}}=\frac{\left|\left\{\left(u,v\right):\left(u,v\right)\in E\wedge y_{u}=y_{v}\right\}\right|}{\left|E\right|},$$ (9)

where E is the set of edges, and $y_{u}$ is the class of node u (Zhu et al., 2020). We designed a pipeline that analyzes scRNA-seq data, utilizes the data to construct undirected graphs, and feeds the graphs into GNN models. The count matrices consist of genes as columns and cells as rows. The values in the matrices show Unique Molecular Identifier (UMI)-filtered counts per cell detected in the raw data. We used LIANA (Türei et al., 2022), a comprehensive toolkit used for predicting L-R interactions, to calculate the communication between individual cells. The interaction can be interpreted as the connectivity of cells to form adjacency matrices. In an adjacency matrix, A = ${\left(a_{ij}\right)}_{N\times N}$ , the values $a_{ij}$ represent cell connectivity and N is the number of cells. Based on the matrix A , we constructed an undirected graph, G = (V,E) , where V is a set of vertices that can be interpreted as cells, and E is a set of paired vertices that can be interpreted as cell connectivity. Given a set of undirected graphs with different H ratios, we aim to predict which cell type the unmarked cells belong to and find out how well the GNN models handle the graphs with low H ratios.

**FIGURE 2:**
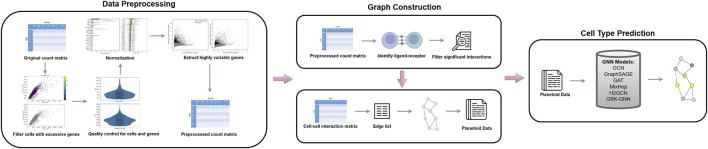
A pipeline for cell type prediction using GNNs. The process begins with data preprocessing, where the original count matrix is normalized, quality control is performed on cells and genes, and highly variable genes are extracted to produce a preprocessed count matrix. Next, graph construction identifies L-R interactions, filters significant interactions, and generates a CCC matrix, which is converted into a graph structure with an edge list formatted as Planetoid data. Finally, various GNN models (e.g., GCN, GraphSAGE, GAT, MixHop, ${\text{H}}_{2}$ GCN, and GBK-GNN) are applied to predict cell types based on the generated graph.

**FIGURE 3:**
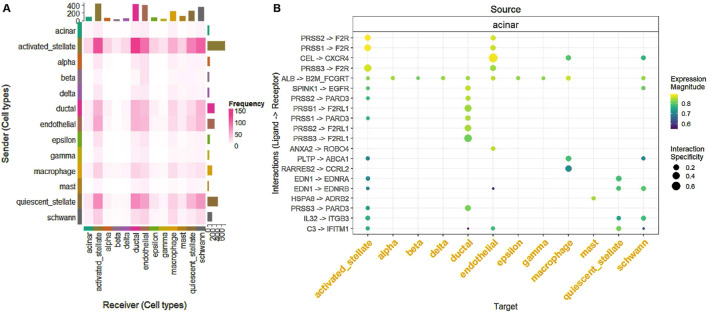
**(A)** Heatmap showing the frequency of L-R interactions between sender (rows) and receiver (columns) cell types. Each cell represents the interaction frequency, with darker shades of pink indicating higher interaction frequencies. This analysis highlights the communication dynamics among various cell types, such as acinar, activated stellate, and endothelial cells. **(B)** Dot plot illustrating specific L-R interactions for each source cell type (rows) and their target cell types (columns). The size of the dots represents the specificity of the interaction, while the color indicates the expression magnitude, providing a detailed overview of prominent L-R pairs and their interaction patterns.

We proposed a pipeline that consists of three main steps: 1) Data preprocessing (Figure 2), 2) Graph construction (Figure 2), and 3) Model training (Figure 2). Data preprocessing is a critical technique of organizing and cleaning raw data to make the data suitable for downstream application and model training. After data is processed, adjacency matrices are calculated along with H ratios, and graphs are derived from adjacency matrices. In the model training step, we converted graphs to a framework called Predicting Labels And Neighbors with Embeddings Transductively Or Inductively from Data (Planetoid) (Cohen et al., 2016), which has been widely used as an input data format of GNNs. The details of implementation are explained in the next sections.

### 2.3 Data preprocessing

Before delving into the analysis of scRNA-seq data, preprocessing the data to mitigate the impact of noise present in the samples is a crucial step. In this regard, we adhered to a standard preprocessing pipeline commonly employed in scRNA-seq data analysis. This comprehensive step encompasses quality control, normalization, and feature selection as illustrated in the initial stage of the pipeline represented in Figure 2. We utilized Scanpy (AngererWolf and SCANPY, 2018), a specialized toolkit designed for the analysis of scRNA-seq data. Scanpy (AngererWolf and SCANPY, 2018) offers a comprehensive suite of functionalities, including data preprocessing, visualization, clustering, differential expression analysis, and the simulation of gene regulatory networks. In contrast, other platforms such as Seurat (Farrell et al., 2015; Hoffman et al., 2018; Stuart et al., 2019; Hao et al., 2021; Hao et al., 2023), R-based Bioconductor (Huber et al., 2015), and Cell Ranger (Zheng et al., 2017) struggle to handle extremely large datasets, particularly those exceeding one million cells. Scanpy (AngererWolf and SCANPY, 2018) effectively overcomes these scalability challenges while maintaining the capability to perform similar analytical tasks. Additionally, it provides a user-friendly interface that integrates seamlessly with advanced machine learning libraries.

The evaluation of data quality in scRNA-seq analysis involves two main components: cell quality control and gene quality control. Common criteria for assessing cell and gene quality include the proportion of counts attributed to mitochondrial reads, the number of total counts per cell, and the number of expressed genes per cell. The analysis of these criteria involves examining their distributions to identify outlier peaks, which can be effectively managed through thresholding. These outlier data points are often associated with various issues such as dying cells, cells with compromised membranes, or doublets. For example, cells characterized by a low number of total counts, a limited number of detected genes, and a high fraction of mitochondrial counts may suggest cells where cytoplasmic mRNA has leaked through a broken membrane, with only conserved mRNA remaining in the mitochondria. By observing the scatter plot of mitochondrial reads percentage in Baron-human, we removed the cells whose mitochondrial reads are greater than 5%. Conversely, cells with unexpectedly high total counts and a large number of detected genes may indicate doublets. As a standard practice, high total-count thresholds are commonly applied to filter out potential doublets and other undesirable outliers. In the dataset Baron-human1, we filtered out 31 cells that have more than 14,000 counts and kept 1,922 cells. As for genes, we filtered out 7,950 genes that were detected in less than 20 cells and kept 12,175 genes.

Normalization aims to standardize the raw count data to remove sampling effects by bringing it to a common scale without altering values or losing information. More specifically, a number of mRNA molecules in the cells cannot be fully captured, resulting in a variation in the total counts detected among cells. We used Counts Per Million (CPM) derived from bulk expression analysis, which utilizes a normalization method that adjusts count data by applying a size factor $10^{6}$ to the total counts per cell. Then, we applied log(1+x) transformation to reduce the skewness of the data to approximate the assumption of many downstream analysis tools that the data are normally distributed (Theis and Luecken, 2019).

Even after removing genes with a low number of counts during the QC step, the dimension of feature space in a scRNA-seq dataset is still beyond 12,000. To mitigate data noise and enhance data visualization, we utilized feature selection to diminish the dataset’s dimensionality. During this phase, the dataset undergoes filtering to retain only informative genes that represent the variability of the data. Therefore, Highly Variable Genes (HVGs) are frequently employed for this purpose. We extracted the 2,000 most variable genes for downstream analysis. The feature matrix X consists of 2,000 selected genes, forming the input for model training. The decision to use 2,000 genes follows standard practice in scRNA-seq analysis, where highly variable genes are prioritized to improve model performance while reducing dimensionality. By focusing on the most informative features, we enhance the model’s ability to discern meaningful patterns without introducing excessive computational costs. The labels y used for model training correspond to cell-type annotations derived from expert-curated datasets. These labels serve as ground truth for node classification tasks, enabling us to assess the ability of different GNN architectures to correctly predict cell identities. Given the heterogeneity in cell types, leveraging these biologically informed labels allows for a more realistic evaluation of model performance.

### 2.4 Graph Construction

To construct graphs, LIANA (Türei et al., 2022) is used to infer L-R interactions between cells, leveraging cell-type information from the metadata. It integrates multiple methods (such as CellPhoneDB (Vento-TormoEfremova et al., 2020), NATMI (Ramilowski et al., 2015; Denisenko et al., 2020), and others) to provide a robust analysis of CCC based on transcriptomics data. We designed a workflow that uses LIANA (Türei et al., 2022) to load gene expression data and metadata, convert them into Seurat (Hoffman et al., 2018; Hao et al., 2021; Hao et al., 2023; Farrell et al., 2015; Stuart et al., 2019) objects, and infer L-R interactions. Table 2 and Figure 3 present key metrics that evaluate the strength, specificity, and statistical significance of each interaction, including rankings and scores from multiple analytical tools.

**TABLE 2:** Summary of significant L-R interactions identified by LIANA.

Source	Target	Ligand complex	Receptor complex	Aggregate rank	Mean rank	Natmi.edge specificity	Natmi rank	Connectome. Weight_sc	Connectome rank	Logfc comb	Logfc rank	Sca.LR score	Sca rank	Cellphonedb P-value	Cellphonedb rank
activated_stellate	endothelial	COL1A1	CD93	$2.13\times 10^{-10}$	1056	0.510	23	3.84	37	5.74	1	0.919	6	0	5216
activated_stellate	endothelial	COL1A2	CD93	$3.88\times 10^{-10}$	1066	0.494	26	3.76	40	5.05	7	0.907	43	0	5216
alpha	mast	GCG	ADRB2	$8.79\times 10^{-8}$	1112	0.256	138	4.00	28	4.99	9	0.897	167	0	5216
macrophage	endothelial	CXCL8	KDR	$2.07\times 10^{-7}$	1105	0.536	22	3.61	65	4.87	14	0.895	207	0	5216
epsilon	quiescent_stellate	GHRL	PTGIR	$2.49\times 10^{-7}$	1664	0.454	37	5.19	4	5.39	2	0.819	3063	0	5216
activated_stellate	endothelial	MMP2	PECAM1	$2.74\times 10^{-7}$	1111	0.402	49	3.66	58	4.97	11	0.894	222	0	5216

The algorithm begins by extracting a set of unique L-R pairs, denoted as P , to ensure comprehensive inclusion of potential interaction candidates. Next, the algorithm retrieves the gene expression data E and sets a predefined threshold T . This threshold acts as a criterion to distinguish biologically meaningful expression levels from background noise, providing a foundation for subsequent analyses. Two matrices, L and R , are initialized to represent the expression profiles of ligands and receptors, respectively. L will hold binary indicators of ligand expression across cells, while R will store binary indicators of receptor expression, with particular attention paid to receptors that comprise multiple subunits.

The algorithm then iteratively processes each L-R pair i from the set P . For each pair, it extracts the ligand l and the receptor r . If the ligand l is present in the expression data E , its expression across all cells is compared to the threshold T , and a binary result is stored in the corresponding row of L . When handling receptors, the algorithm differentiates between single-subunit and multi-subunit receptors. If r is a multi-subunit receptor, it will be split into subunits separated by an underscore symbol. The algorithm checks whether all subunits are present in E , and only cells expressing all subunits above the threshold T are recorded in R[i,:] . If r is a single-subunit receptor present in E , the expression profile is similarly compared to T and recorded in R[i,:] .

To construct the weighted interaction matrix W , the algorithm initializes W with zeros, and then iterates over each source cell s to compute interaction scores. For each source cell, the ligand expression profile $L_{s}$ is retrieved, and the interaction score $S_{j}$ for every target cell j is calculated. This score is obtained by summing the products of $L_{s}\left[i\right]$ and R[i,j] across all L-R pairs i , representing the interaction strength between s and j . The interaction scores are used to update the corresponding row of W . Afterward, W is normalized by dividing each element by the maximum value in the matrix, which standardizes the interaction scores. A threshold is then applied to retain only the top-weighted interactions, which highlights significant connections between cells.

To ensure the connectivity of the inferred cell communication network, the algorithm performs a refinement step to examine each node i in W and checks for isolated nodes, that have no outgoing or incoming connections. If such a node is identified, the algorithm connects it to the cell with the highest interaction weight, ensuring the network remains biologically plausible and connected. Finally, the refined adjacency matrix W , which represents the complete CCC graph, is returned as the output of the algorithm. This structured approach ensures that the inferred network captures meaningful cellular interactions while maintaining connectivity. We computed the edge homophily ratio (H) for each dataset to quantify the extent of homophilic *versus* heterophilic connections. The homophily ratio serves as a key metric in our study, allowing us to systematically compare the performance of different models across graphs with varying structural properties. By incorporating ligand-receptor interactions inferred using LIANA, we ensured that our graphs capture biologically meaningful cell-cell communication pathways. This biologically grounded graph construction differentiates our approach from conventional GNN benchmarks, which often rely on artificially defined connectivity patterns.

Algorithm 1 CCC Network Construction.

**procedure** Cell-Interaction-Graph ( P,E,T )
   Extract unique L-R pairs P

   Retrieve expression data E and set threshold T

   Initialize L : Ligand expression matrices
   Initialize R : Receptor expression matrices
   **for** each i∈P **do**
     
l← ligand of i , r← receptor of i

     **if** l∈E **then**
         
L[i,:]←E[l,:]>T

     **end** **if**
     **if** r is multi-subunit **then**
         Split r into subunits
         **if** all subunits ∈E **then**
           
R[i,:]← cells expressing all subunits above T

         **end** **if**
     **else** **if** r∈E **then**
         
R[i,:]←E[r,:]>T

     **end** **if**
   **end** **for**
   Initialize W←0 : weighted interaction matrix
   **for** each source cell s **do**
     
$L_{s}\leftarrow L\left[,s\right]$

     
$S_{j}\leftarrow \sum_{i}\left(L_{s}\left[i\right]\cdot R\left[i,j\right]\right)\;\forall j$

     Update W[s,:] with $S_{j}$

   **end** **for**
   Normalize W←W/max(W)

   Apply threshold to top weights in W

   **for** each i∈W **do**
     **if** W[i,:] and W[:,i] have no connections **then**
       Connect i to cell with max weight
     **end** **if**
   **end** **for**
   **return** W

**end** **procedure**

### 2.5 Model training

Planetoid (Cohen et al., 2016) is a graph embedding framework focusing on semi-supervised learning, which provides a paradigm of the input data for both transductive and inductive models. Since it has been used as the benchmark for evaluating the performance of GNNs, as explained in Graph Construction section, we converted the adjacency matrices to eight objects that can be directly plugged into inductive GNN models.

• **X** contains the feature vectors of the labeled training instances. We selected 140 cells as the train instances represented in the Compressed Sparse Row matrix.

• **y** contains the one-hot labels of the training instances X.

• **allX** is a superset of X, which contains the feature vectors of both labeled and unlabeled training instances.

• **ally** is a superset of y, which contains labels for instances in allX.

• **tX** contains the feature vectors of the test instances. We selected 1,000 cells as the test instances represented in the Compressed Sparse Row matrix.

• **ty** contains the one-hot labels of the training instances tX.

• **graph** is a dictionary in the format {index-of-cells [index-of-neighbor-cells]}.

• **test. index** contains the indices of test instances in graph (Cohen et al., 2016).

#### 2.5.1 Train-test splitting strategy

We followed the widely used “Planetoid” split protocol, selecting 140 training cells (10 per class) while reserving 1,000 cells for testing. This approach aligns with prior transductive GNN benchmarks and reflects real-world single-cell scenarios, where labeled annotations are scarce. The test set size was chosen to ensure statistical robustness while maintaining a consistent evaluation protocol across datasets. In addition to the Planetoid-style split (140 training cells), we conducted experiments using an 80/20 train-test split, where 80% of the available cells were used for training and 20% for testing. This comparison allowed us to evaluate the impact of training set size on model performance and assess the adaptability of heterophily-aware GNNs to different training regimes. Extended results using an 80/20 split are available in Supplementary Table S2.

#### 2.5.2 Hyperparameter selection

To ensure optimal model performance, we performed hyperparameter tuning for all models. We utilized grid search and empirical tuning to determine the best learning rate, dropout rate, weight decay, and architecture-specific parameters. Supplementary Table S1 in the supplementary materials provides a summary of the hyperparameters used for each model. For instance, we found that GBK-GNN performed best with a learning rate of 0.001, while MixHop required a higher dropout rate (0.7) to prevent overfitting. Additionally, deeper architectures such as ${\text{H}}_{2}$ GCN and GBK-GNN required extended training epochs (up to 2000) to converge. These choices were made based on prior literature and iterative experiments to balance model accuracy and computational efficiency.

#### 2.5.3 Multiple seed experiments and reproducibility

**FIGURE 4:**
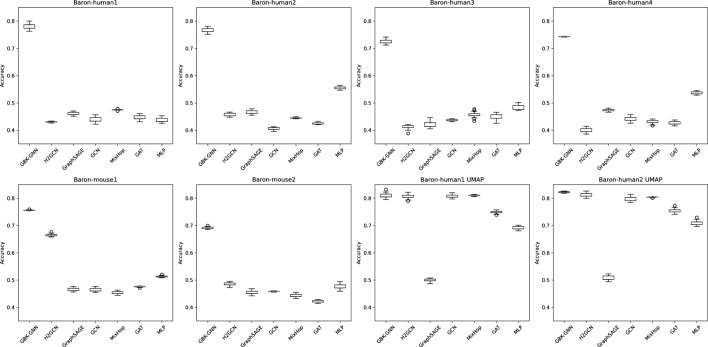
Boxplots illustrating the accuracy distribution of different graph neural network (GNN) models across 30 random seed runs for multiple datasets. Each subplot corresponds to a different dataset, and within each plot, the boxplots represent the performance variability of different models. The x-axis denotes the evaluated models, while the y-axis represents classification accuracy. Wider interquartile ranges and the presence of outliers indicate models with higher sensitivity to seed initialization. Models demonstrating tight boxplots exhibit greater stability across different training runs. These results highlight the importance of evaluating performance over multiple seeds to ensure the reproducibility and robustness of conclusions.

To ensure the robustness and stability of our findings, we evaluated model performance across multiple random initializations by running each experiment with 30 different random seeds (see Supplementary Tables S3–S10). This approach allows us to assess the stability of our conclusions and provides insight into how heterophily-aware Graph Neural Networks (GNNs) perform in diverse biological settings. Boxplots illustrating accuracy distributions across different seeds were generated, as shown in Figure 4.

## 3 Results

### 3.1 Impact of random seed variability

To ensure that performance comparisons are not biased by a specific train-test partition, we trained each model across 30 different random seeds. Figure 4 presents boxplots illustrating accuracy distributions, confirming that heterophily-aware models consistently outperform baseline GNNs with minimal variance across different random initializations. These results emphasize the importance of evaluating models under diverse sampling conditions to assess their true robustness.

### 3.2 Effectiveness of heterophily-aware models

Table 4 summarizes the classification accuracy across datasets with different homophily ratios (H) . GBK-GNN consistently outperforms other models, particularly in low- H settings, demonstrating its robustness in heterophilic graphs. These findings highlight the necessity of specialized architectures for analyzing biological networks, where interactions frequently occur between dissimilar entities. For performance comparison, we selected a set of baseline models comprising MLP, GCN, GraphSAGE, GAT, and MixHop, along with two advanced models designed specifically for heterophily: ${\text{H}}_{2}$ GCN and GBK-GNN. To ensure consistency and reproducibility, we utilized the original source code from publicly accessible GitHub repositories. All default parameters, such as the number of epochs and the train-test split ratio, were retained for each model during the evaluation process.

We evaluated the computational efficiency of GNN models by comparing training time per epoch, inference time, and memory usage (Table 3). GCN and MLP were the most efficient, requiring minimal computational resources, making them ideal for large-scale applications with hardware constraints. In contrast, GBK-GNN and ${\text{H}}_{2}$ GCN, while achieving higher accuracy, had longer training times due to their complex architectures. GraphSAGE and GAT provided a balanced trade-off between efficiency and performance, making them suitable for resource-aware applications.

**TABLE 3:** Computational efficiency of each GNN model on the Baron-human one dataset for 2000 epochs. The complexity is expressed in Big O notation, where e denotes the number of edges, n the number of nodes, d the dimensionality of node embeddings, L the number of layers, h the number of hops considered (for hop-based models like ${\text{H}}_{2}$ GCN), k the number of adjacency matrices (in MixHop), and $h_{a}$ the number of attention heads (specific to GAT). This notation highlights how resource usage scales with various network and graph parameters, with RAM usage reflecting space complexity and execution time capturing both training and validation durations.

Metric Model	GBKGNN	${\text{H}}_{2}$ GCN	GraphSAGE	GCN	MixHop	GAT	MLP
Execution Time (HH:MM:SS)	00:09:40	00:04:39	00:10:48	00:06:13	00:04:05	00:03:16	00:05:48
RAM Usage (GB)	2.3	2.4	2.4	2.1	2.4	1.8	2.1
GPU Usage (GB)	37.6	5.7	1.6	7.2	1.5	2.9	9.7
RAM Usage Complexity	O(e)	O(e)	O(e)	O(e⋅d)	O(e)	O(e)	O(n⋅d)
Execution Time Complexity	O(L(e⋅d))	O(L(h⋅e⋅d))	$O\left(L\left(n\cdot d^{2}\right)\right)$	$O\left(L\left(n\cdot d^{2}\right)\right)$	O(L(k⋅e⋅d))	$O\left(L\left(e\cdot d\cdot h_{a}\right)\right)$	$O\left(L\left(n\cdot d^{2}\right)\right)$
Inference Time Complexity	O(L(e⋅d))	O(L(h⋅e⋅d))	$O\left(L\left(n\cdot d^{2}\right)\right)$	$O\left(L\left(n\cdot d^{2}\right)\right)$	O(L(k⋅e⋅d))	$O\left(L\left(e\cdot d\cdot h_{a}\right)\right)$	$O\left(L\left(n\cdot d^{2}\right)\right)$

The comparison of GNN models and MLP performance across various datasets, as presented in Table 4, highlights key insights into the adaptability of heterophily-aware methods for real-world datasets with high heterophily (low H ratio). Since most datasets in this study exhibit high heterophily, we utilized the Baron-human1 and Baron-human2 datasets to construct two additional graphs using UMAP (McInnes et al., 2018) with a high H ratio. By specifying a neighborhood size and distance metric, we generated a fuzzy simplicial set represented as a sparse matrix corresponding to the input data. This process included estimating geodesic distances for each data point, constructing fuzzy simplicial sets for individual points, and combining these sets into a global representation via a fuzzy union (McInnes et al., 2018). Through this methodology, we derived graphs with varying distance metrics and neighborhood sizes, achieving H ratios of 0.83 and 0.88, as shown in Table 4.

**TABLE 4:** Accuracy comparison of models across various scRNA-seq datasets. Best model per dataset highlighted in gray.

Model Dataset	Baron-human1	Baron-human2	Baron-human3	Baron-human4	Baron-mouse1	Baron-mouse2	Baron-human1 UMAP	Baron-human2 UMAP
**Hom. ratio** h	0.15	0.26	0.22	0.21	0.26	0.27	0.83	0.88
#Nodes |V|	1,904	1,713	3,592	1,251	761	913	1,904	1,713
#Edges |E|	510,456	515,956	2,214,355	276,193	106,559	216,151	122,088	186,250
#Classes |Y|	14	14	14	14	13	13	14	14
**GBK-GNN**	0.781	0.765	0.724	0.743	0.756	0.691	0.812	0.823
**H**_**2**_ **GCN**	0.430	0.459	0.412	0.400	0.664	0.486	0.808	0.814
**GraphSAGE**	0.462	0.467	0.419	0.472	0.464	0.455	0.500	0.509
**GCN**	0.439	0.405	0.437	0.442	0.465	0.458	0.808	0.797
**MixHop**	0.474	0.446	0.457	0.431	0.453	0.446	0.810	0.804
**GAT**	0.446	0.424	0.453	0.427	0.475	0.422	0.749	0.756
**MLP**	0.439	0.554	0.483	0.536	0.512	0.476	0.694	0.709

## 4 Discussion

### 4.1 Heterophily-aware methods

• $H_{2}$ **GCN:** $H_{2}$ GCN underperforms compared to other models on most datasets with high heterophily, and this can be partially attributed to the structural characteristics of the datasets, such as the number of edges and nodes. For instance, in datasets Baron-human3 (|V|=3,592,|E|=2,214,355,h=0.22) and Baron-human4 (|V|=1,251,|E|=276,193,h=0.21) , the model achieves relatively low accuracies of 0.412 and 0.400, respectively. These datasets have large numbers of edges relative to the number of nodes, resulting in high average node degrees. In high heterophily settings, this dense connectivity can lead to noise in the neighborhood aggregation process, as connected nodes are more likely to belong to different classes. $H_{2}$ GCN’s design, which relies on higher-order neighborhoods and aggregating information from multiple hops, may worsen this issue by propagating irrelevant or conflicting information in such environments.

Additionally, the sparse graph structure in smaller datasets such as Baron-mouse2 (|V|=913,|E|=216,151,h=0.27) further contributes to $H_{2}$ GCN’s limitations, where it achieves an accuracy of 0.486. Sparse connectivity can limit the availability of meaningful node relationships for effective aggregation, particularly in highly heterophilic settings where even the few existing edges are less likely to connect nodes of the same class. These attributes of the datasets—dense or sparse edge distributions and high heterophily—reduce the effectiveness of $H_{2}$ GCN’s reliance on structural features and its neighborhood propagation mechanism, leading to its underperformance compared to models better equipped to handle heterophilic graphs, such as GBK-GNN.

• **GBK-GNN:** GBK-GNN demonstrates consistently strong performance across datasets, outperforming other models in both heterophilic and UMAP-modified settings. For example, on Baron-human3 (h=0.22) and Baron-human4 (h=0.21) , GBK-GNN achieves accuracies of 0.724 and 0.743, significantly higher than those of $H_{2}$ GCN. On the graphs constructed by UMAP with h=0.83 and h=0.88 , GBK-GNN further excels with accuracies of 0.812 and 0.823. This robust performance highlights GBK-GNN’s ability to effectively capture both local and global graph structures, making it well-suited for datasets with varying levels of heterophily.

### 4.2 Performance comparison with homophily-assuming methods

• Models like **GCN, GraphSAGE, and GAT**, which typically assume homophily, show a noticeable increase in performance at higher H ratios but fall short in low H ratio scenarios. On Baron-human3 (h=0.22) and Baron-human4 (h=0.21) , these models achieve relatively low accuracies, with GraphSAGE scoring 0.419 and 0.437, GCN achieving 0.412 and 0.437, MixHop at 0.457 and 0.443, and GAT at 0.427 and 0.435. These results reveal their struggle to effectively learn node representations in heterophilic graphs where connected nodes often belong to different classes. GraphSAGE’s reliance on aggregating information from immediate neighbors leads to noise propagation in such settings, while GCN and GAT similarly depend on local neighborhood aggregation, making them less robust to the lack of homophilic structure. MixHop, which leverages higher-order neighborhood information, shows slightly better performance but still suffers from propagating conflicting signals in dense heterophilic graphs. These models generally assume that neighboring nodes share similar labels, which limits their ability to distinguish between dissimilar nodes in highly heterophilic graphs, contributing to their relatively low performance.

• The **MLP** model shows competitive performance on some heterophilic datasets, such as Baron-human2 (h=0.26) with an accuracy of 0.554, but struggles on others like Baron-human3 (h=0.22) with an accuracy of 0.483. Unlike graph-based models, MLP does not rely on graph structure and instead focuses solely on the node features for prediction. This independence from graph connectivity makes MLP less susceptible to the noise introduced by heterophilic edges, allowing it to perform well on datasets where node features are more informative. This result reflects MLP’s reliance on feature quality over structural insights, making it less robust in scenarios where the graph structure is essential for capturing relationships between nodes.

In summary, GBK-GNN consistently outperforms traditional homophily-assuming GNNs and $H_{2}$ GCN in handling datasets with high heterophily, demonstrating robustness and adaptability to diverse graph structures. $H_{2}$ GCN performed poorly due to its reliance on higher-order neighborhood aggregation, which amplifies noise in densely connected heterophilic graphs and struggles with sparse connectivity in smaller datasets. The bi-kernel mechanism and gate selection in GBK-GNN enables it to dynamically adapt to varying levels of homophily and heterophily, achieving significantly higher accuracies across diverse datasets. These findings underline GBK-GNN’s effectiveness for complex real-world applications, such as computational biology, where graph structures are heterogeneous and the homophily assumption often fails.

### 4.3 Biological assessment

Computational methods such as LIANA (Türei et al., 2022) predict cell-cell connections by identifying L-R interactions, but these predictions need to be biologically verified to ensure their reliability and relevance. CellCall (Zhang et al., 2021) is an advanced computational tool designed to illuminate and validate intercellular communications within scRNA-seq datasets. By integrating gene expression profiles with known L-R interactions, CellCall (Zhang et al., 2021) systematically infers and assesses the significance of CCC pathways, thereby providing valuable insights into the complex biological interactions that underpin various physiological and pathological processes. The workflow commences with the preparation of expression data and corresponding metadata extracted from a Seurat (Farrell et al., 2015; Hoffman et al., 2018; Stuart et al., 2019; Hao et al., 2021; Hao et al., 2023) object, a widely adopted framework for single-cell analysis. This data is subsequently encapsulated within a CellCall (Zhang et al., 2021) object, which serves as the foundation for further analyses.

The core functionality of CellCall (Zhang et al., 2021) involves the profiling of intercellular communications through the TransCommuProfile function, which applies statistical thresholds to identify significant L-R interactions based on correlation and p-value criteria. Following this, the method employs hypergeometric testing to determine the enrichment of specific signaling pathways within the identified interactions, thereby highlighting biologically relevant communication routes.

**FIGURE 5:**
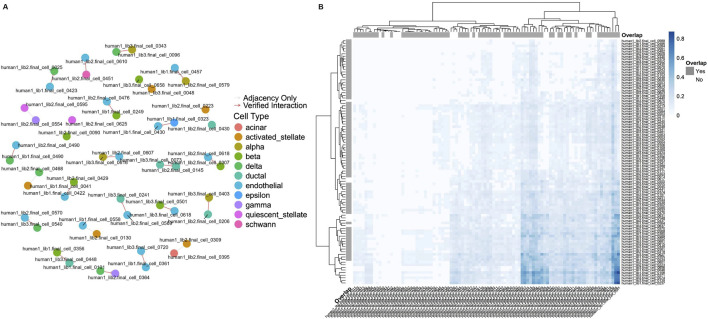
**(A)** Network graph illustrating cell-cell interactions as derived from the CellCall pipeline. Nodes represent individual cells, color-coded by their assigned cell type, and edges indicate interactions. Verified L-R interactions are shown in red, while gray edges represent adjacency-only connections. The graph highlights the overlap between adjacency-based cell relationships and those validated through significant signaling pathways. **(B)** Hierarchical clustering heatmap illustrating the connection scores between cells as inferred by LIANA. Rows and columns represent individual cells, with color intensity indicating the strength of the connection, where darker blue shades correspond to higher connection scores. Overlap annotations (“Yes” or “No”) indicate whether the connections were verified by CellCall.

To validate the inferred cell-cell pairs, CellCall (Zhang et al., 2021) cross-references the significant signaling pathways with an adjacency matrix of direct cell interactions, quantifying the overlap and thereby assessing the reliability of the predicted communications. This comprehensive approach not only identifies potential CCC but also substantiates them through statistical validation, ensuring that the findings are both robust and biologically meaningful. We selected the Baron-human1 dataset as an example, and 72% of the cell-cell connections in the adjacency matrix are part of the significant signaling pathways. The visualized results are in Figure 5. To further validate the biological significance of our predicted cell-cell interactions, we mapped a subset of ligand-receptor pairs onto Reactome pathways, illustrating their involvement in well-characterized signaling cascades. Supplementary Figure S3 highlights key interactions, including the SEMA4D-CD72 pair, which plays a role in B cell receptor (BCR) signaling. SEMA4D, a membrane-bound semaphorin expressed on T cells, binds to CD72 on B cells, modulating B cell activation and costimulatory signaling. The highlighted edges in Supplementary Figure S3 illustrate this interaction within the broader immune signaling network. Additionally, we filtered and ranked ligand-receptor pairs using NicheNet, prioritizing interactions with high signaling relevance. Supplementary Figure S1 presents a bar plot summarizing the signaling weights of key ligand-receptor pairs, where higher values indicate stronger biological significance. This ranking provides a quantitative assessment of interaction strength, helping identify the most functionally relevant connections. Finally, we visualized the ligand-receptor interaction network in Supplementary Figure S2, where nodes represent ligands and receptors, and edge width reflects signaling strength. This network analysis provides an integrated view of cell-cell communication pathways, emphasizing key interactions with strong biological relevance. These findings reinforce the applicability of heterophily-aware GNNs in modeling complex biological interactions and suggest their potential for broader transcriptomic studies. While this study focuses on pancreatic islet datasets, our methodology is broadly applicable to other single-cell transcriptomic datasets, including immune cells, brain tissue, and tumor microenvironments, where cell-cell communication plays a crucial role. Future work will assess the robustness of heterophily-aware GNNs across diverse biological contexts. Additionally, we constructed graphs based solely on ligand-receptor interactions, which provide a biologically meaningful foundation; however, integrating other interaction patterns, such as transcription factor-target gene relationships and metabolic dependencies, could further enhance predictive performance. Expanding graph construction strategies and testing the models on more diverse datasets will strengthen the applicability and biological relevance of our approach.

## 5 Conclusion

This study underscores the transformative potential of heterophily-aware GNNs in the analysis of scRNA-seq data within computational biology. Traditional GNN models operate under the homophily assumption—where nodes with similar characteristics are more likely to be interconnected. However, our investigation reveals that biological networks often exhibit high heterophily, where dissimilar nodes frequently interact, challenging the efficacy of these conventional models.

To address this complexity, we employed LIANA (Türei et al., 2022) to construct biologically informed interaction graphs based on L-R pairs. LIANA (Türei et al., 2022) integrates multiple established methods to infer cell-cell communication pathways, generating adjacency matrices that more accurately reflect the underlying biological interactions. This robust graph construction facilitated the application of advanced GNN models such as GBK-GNN (Du et al., 2022) and $H_{2}$ GCN (Zhu et al., 2020), specifically designed to handle heterophilic relationships.

The experimental results demonstrate that heterophily-aware models, particularly GBK-GNN, significantly outperform traditional GNNs and even non-graph-based models like MLP across various scRNA-seq datasets. GBK-GNN’s dual-kernel mechanism and dynamic gating system enable it to effectively differentiate and integrate both homophilic and heterophilic connections, leading to superior predictive performance. In contrast, models like $H_{2}$ GCN (Zhu et al., 2020), despite being designed for heterophilic data, underperform in densely or sparsely connected biological graphs due to their reliance on higher-order neighborhood aggregations, which introduce noise and reduce model accuracy.

Further validating our findings, biological assessment using CellCall (Zhang et al., 2021) confirmed the reliability of the inferred cell-cell communication pathways. CellCall’s integration of L-R interactions with transcription factor activities revealed that a substantial proportion of predicted interactions align with known biological mechanisms, reinforcing the biological relevance of our graph constructions and GNN predictions. This validation highlights the capability of heterophily-aware GNNs to not only excel in computational performance but also provide meaningful biological insights, thereby bridging the gap between machine learning and biomedical research.

In conclusion, our study not only reaffirms the critical role of GNNs in analyzing structured biological data but also emphasizes the necessity of integrating domain-specific frameworks like LIANA (Türei et al., 2022) and CellCall (Zhang et al., 2021) to enhance biological interpretability. As biological datasets continue to grow in complexity and scale, adopting models that account for the inherent heterogeneity of biological interactions will be essential. By incorporating statistical reproducibility analyses and leveraging biologically informed graphs, our study strengthens the role of heterophily-aware GNNs in computational biology. Future work will explore extending these methods to larger-scale datasets and real-time single-cell analyses.

## Data Availability

The datasets presented in this study can be found in online repositories. The names of the repository/repositories and accession number(s) can be found in the article/Supplementary Material.
