# Supplementary material for: Ligand-receptor dynamics in heterophily-aware graph neural networks for enhanced cell type prediction from single-cell RNA-seq data
Source: Front Mol Biosci. 2025 May 12;12:1547231. doi: 10.3389/fmolb.2025.1547231 (PMC12104675; doi:10.3389/fmolb.2025.1547231)
Supplement: Supplementary file 1 [file Supplementaryfile1.pdf]

Supplementary Material

The Supplementary Material for this article includes additional tables, figures, and experimental results.

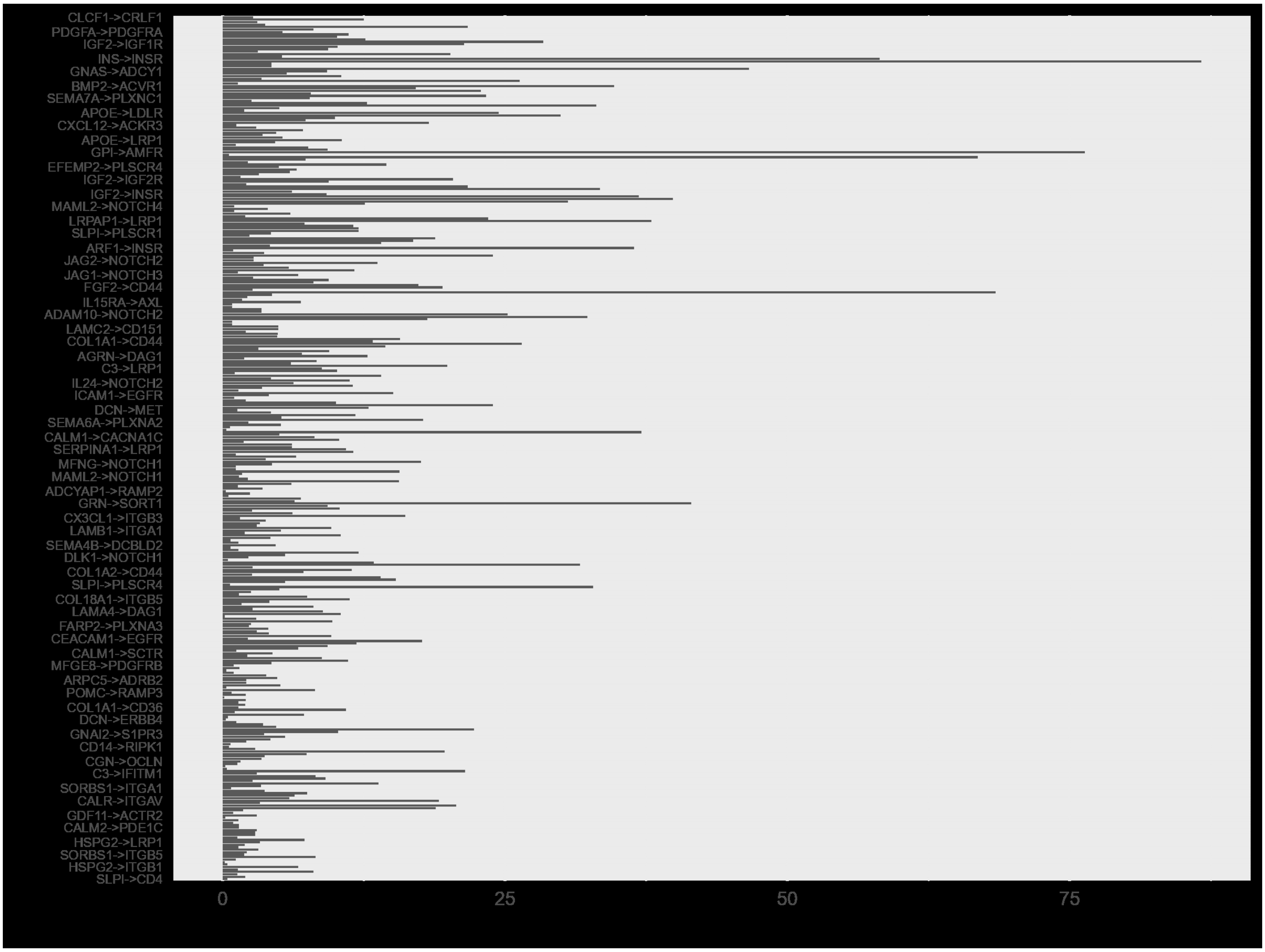

Fig. S1. Bar plot displays the signaling weights associated with each ligand-receptor pair filtered from LIANA using NicheNet's database. Each bar represents a unique LR pair, with bar length indicating the strength of the predicted signaling interaction; higher values suggest more robust biological relevance.

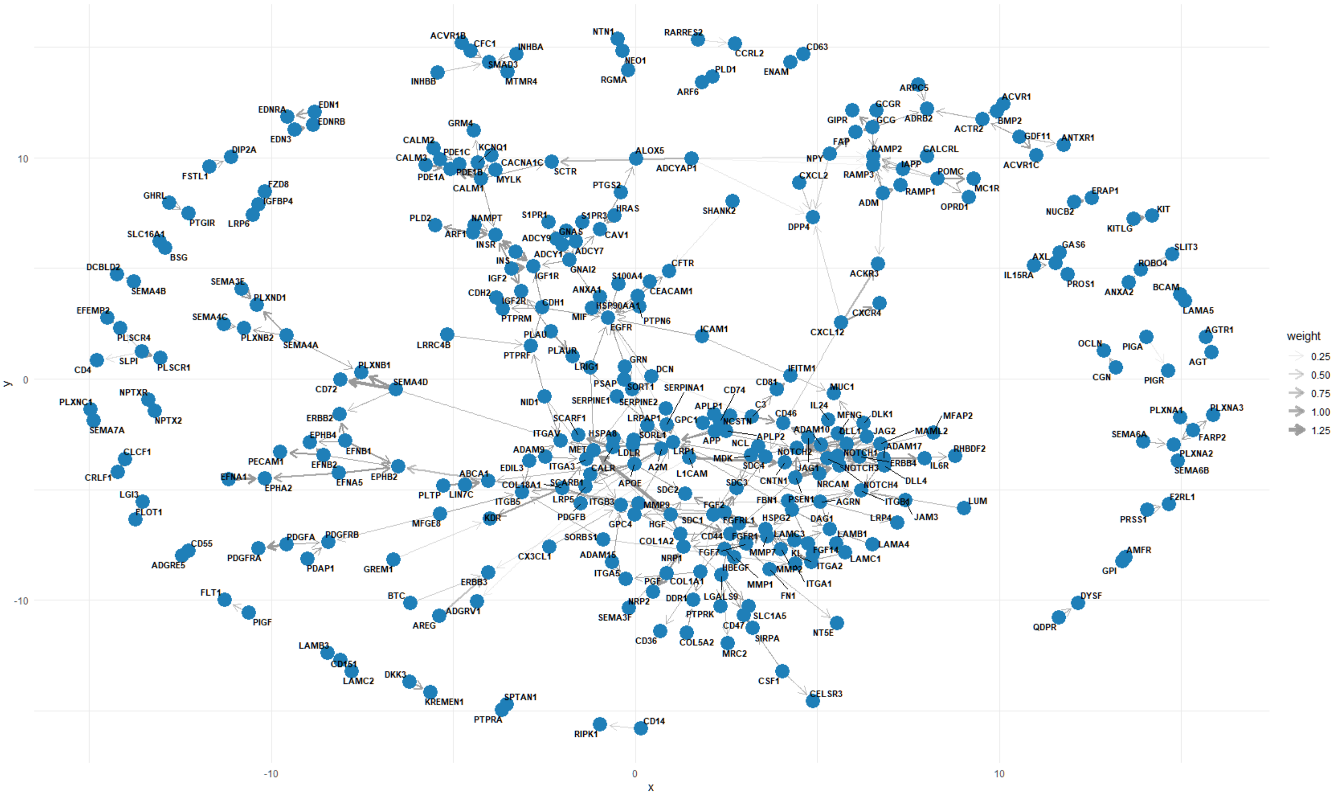

Fig. S2. In the network diagram, nodes represent ligands and receptors, and directed edges indicate the flow of signaling. The edge width and transparency are scaled to the signaling weight, highlighting the most significant interactions. This visualization provides an integrated view of the signaling network, emphasizing the pairs with strong evidence of regulatory connectivity.

Hyperparameters for MLP						
learning_rate	hidden1	dropout	weight_decay	early_stopping	max_degree	epochs
0.01	16	0.5	5e-4	10	3	2000
Hyperparameters for GAT						
learning_rate	l2_coef	hid_units	n_heads	nhood	attn_drop	epochs
0.005	0.0005	8	[8, 1]	1.0	0.6	1500
Hyperparameters for MixHop						
learn_rate	input_dropout	layer_dropout	early_stop_steps	lr_decrement_every	hidden_dims_csv	num_train_steps
0.5	0.7	0.9	50	40	60	400
Hyperparameters for GCN						
learning_rate	hidden1	dropout	weight_decay	early_stopping	max_degree	epochs
0.01	16	0.5	5e-4	10	3	2000
Hyperparameters for GraphSAGE						
learning_rate	hid_units	batch_size	num_samples	epochs		
0.7	128	256	[5, 5]	500		
Hyperparameters for H_2GCN						
epochs						
2000						
Hyperparameters for GBK-GNN						
learning_rate	weight_decay	split	lamda	model_type	dim_size	epochs
0.001	0.01	[0.6, 0.2, 0.2]	1.84	GraphSage	16	500

Table S1. Summary of hyperparameters used by methods.

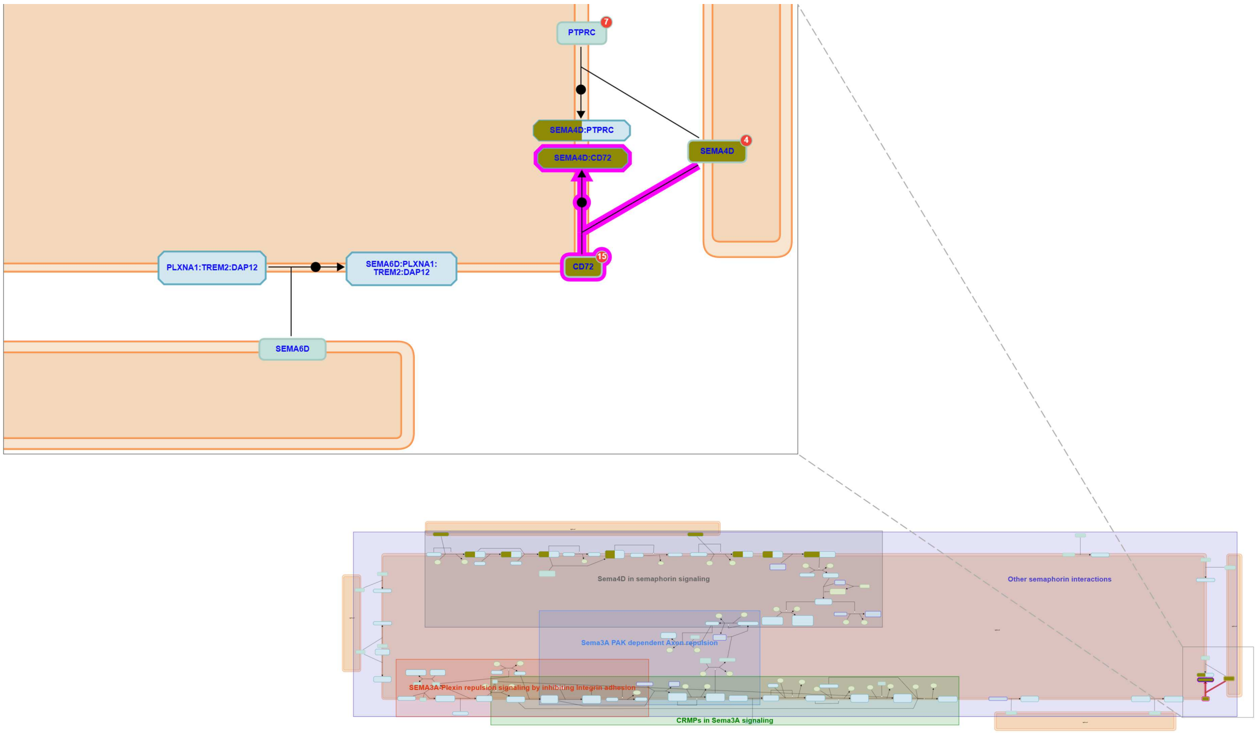

Fig. S3. Mapping of ligand-receptor interactions onto Reactome pathways. The figure highlights key signaling interactions, including the SEMA4D-CD72 pair, which plays a role in B cell receptor (BCR) signaling. SEMA4D, expressed on T cells, binds to CD72 on B cells, modulating B cell activation and costimulatory signaling. The highlighted edges indicate direct interactions, while the colored boxes represent distinct signaling pathways.

	**Baron-human1**	**Baron-human2**	**Baron-human3**	**Baron-human4**	**Baron-mouse1**	**Baron-mouse2**	**Baron-human1 UMAP**	**Baron-human2 UMAP**
**GBK-GNN**	0.7589	0.7466	0.7384	0.7523	0.5423	0.5244	0.7984	0.8087
**H_2GCN**	0.4961	0.5598	0.5279	0.5697	0.7320	0.4372	0.8056	0.8242
**GraphSAGE**	0.462	0.467	0.419	0.472	0.464	0.455	0.500	0.509
**GCN**	0.4316	0.3684	0.4029	0.4400	0.3421	0.4066	0.8034	0.7832
**MixHop**	0.474	0.446	0.457	0.431	0.453	0.446	0.810	0.804
**GAT**	0.4316	0.3684	0.4235	0.4399	0.3421	0.4066	0.7843	0.7690
**MLP**	0.6263	0.7368	0.8324	0.5920	0.8026	0.4066	0.7254	0.7355

Table S2. Accuracy comparison of models across various scRNA-seq datasets using an 80/20 train-test split. Although both GBK-GNN and H_2GCN are designed with heterophilic graphs in mind, their specific mechanisms can perform differently depending on the dataset's structure and the particular train/test split. In some cases (for example, Baron-mouse1), H_2GCN's multi-hop neighbor aggregation aligns better with the graph's connectivity patterns and label distributions, capturing longer-range relationships that are more predictive than the short-range interactions GBK-GNN emphasizes. Meanwhile, GBK-GNN can sometimes struggle if the 80/20 split yields a less representative training subset—which may lead to overfitting—or if its neighborhood selection strategy does not mesh well with the dataset's degree distribution or local clustering. As a result, H_2GCN's focus on higher-order neighborhoods may generalize more effectively under certain data splits and low-homophily conditions, allowing it to outperform GBK-GNN.

**Model**	**Seed 1**	**Seed 2**	**Seed 3**	Seed 4-26	**Seed 27**	**Seed 28**	**Seed 29**	**Seed 30**
GBK-GNN	0.8139	0.8054	0.8100	-	0.8100	0.8037	0.8078	0.8083
H_2GCN	0.8056	0.8069	0.8144	-	0.8073	0.8111	0.7992	0.8163
GraphSAGE	0.4869	0.4904	0.4982	-	0.4994	0.4969	0.4962	0.5014
GCN	0.8162	0.8064	0.8096	-	0.8038	0.8137	0.7975	0.8002
MixHop	0.8119	0.8129	0.8136	-	0.8062	0.8103	0.8104	0.8080
GAT	0.7563	0.7516	0.7572	-	0.7530	0.7459	0.7482	0.7492
MLP	0.6875	0.6971	0.6978	-	0.6885	0.6996	0.6962	0.6803

Table S3. Performance of different GNN models across multiple random seeds on Baron-human1 UMAP dataset.

Model	Seed 1	Seed 2	Seed 3	Seed 4-26	Seed 27	Seed 28	Seed 29	Seed 30
GBK-GNN	0.7953	0.7699	0.7734	-	0.8001	0.7743	0.7818	0.7796
H_2GCN	0.4287	0.4295	0.4311	-	0.4293	0.4304	0.4298	0.4301
GraphSAGE	0.4605	0.4571	0.4682	-	0.4538	0.4665	0.4659	0.4538
GCN	0.4523	0.4489	0.4453	-	0.4421	0.4503	0.4225	0.4336
MixHop	0.4735	0.4727	0.4754	-	0.4735	0.4736	0.4788	0.4743
GAT	0.4479	0.4607	0.4497	-	0.4439	0.4423	0.4530	0.4344
MLP	0.4442	0.4469	0.4412	-	0.4449	0.4256	0.4527	0.4475

Table S4. Performance of different GNN models across multiple random seeds on Baron-human1 dataset.

Model	Seed 1	Seed 2	Seed 3	Seed 4-26	Seed 27	Seed 28	Seed 29	Seed 30
GBK-GNN	0.8230	0.8219	0.8184	-	0.8250	0.8206	0.8246	0.8245
H_2GCN	0.8220	0.8164	0.8032	-	0.8129	0.8178	0.8173	0.7990
GraphSAGE	0.5089	0.5043	0.5121	-	0.5067	0.5151	0.5185	0.5017
GCN	0.8074	0.8020	0.7988	-	0.8032	0.8021	0.7950	0.7889
MixHop	0.8037	0.8014	0.8038	-	0.8012	0.8037	0.8025	0.8036
GAT	0.7539	0.7536	0.7576	-	0.7512	0.7525	0.7576	0.7550
MLP	0.7298	0.7053	0.7066	-	0.7160	0.7006	0.7088	0.7080

Table S5. Performance of different GNN models across multiple random seeds on Baron-human2 UMAP dataset.

Model	Seed 1	Seed 2	Seed 3	Seed 4-26	Seed 27	Seed 28	Seed 29	Seed 30
GBK-GNN	0.7614	0.7564	0.7691	-	0.7688	0.7652	0.7633	0.7772
H_2GCN	0.4542	0.4628	0.4602	-	0.4521	0.4617	0.4594	0.4530
GraphSAGE	0.4693	0.4689	0.4698	-	0.4602	0.4633	0.4622	0.4551
GCN	0.4086	0.4092	0.4113	-	0.4034	0.4010	0.4025	0.4112
MixHop	0.4433	0.4439	0.4441	-	0.4443	0.4438	0.4464	0.4435
GAT	0.4230	0.4274	0.4230	-	0.4233	0.4225	0.4259	0.4233
MLP	0.5584	0.5534	0.5644	-	0.5519	0.5569	0.5580	0.5495

Table S6. Performance of different GNN models across multiple random seeds on Baron-human2 dataset.

Model	Seed 1	Seed 2	Seed 3	Seed 4-26	Seed 27	Seed 28	Seed 29	Seed 30
GBK-GNN	0.7248	0.7135	0.7314	-	0.7247	0.7245	0.7326	0.7204
H_2GCN	0.4165	0.4112	0.4127	-	0.4084	0.4100	0.4154	0.4126
GraphSAGE	0.4367	0.4152	0.4305	-	0.4138	0.4222	0.4134	0.4181
GCN	0.4334	0.4404	0.4384	-	0.4383	0.4374	0.4352	0.4332
MixHop	0.4569	0.4618	0.4775	-	0.4554	0.4570	0.4508	0.4596
GAT	0.4534	0.4573	0.4496	-	0.4257	0.4607	0.4422	0.4460
MLP	0.5012	0.4897	0.4782	-	0.4956	0.4742	0.4726	0.4730

Table S7. Performance of different GNN models across multiple random seeds on Baron-human3 dataset.

Model	Seed 1	Seed 2	Seed 3	Seed 4-26	Seed 27	Seed 28	Seed 29	Seed 30
GBK-GNN	0.7436	0.7425	0.7430	-	0.7439	0.7438	0.7446	0.7433
H_2GCN	0.4145	0.3911	0.3934	-	0.4018	0.4062	0.4083	0.3961
GraphSAGE	0.4719	0.4748	0.4726	-	0.4757	0.4760	0.4769	0.4702
GCN	0.4487	0.4326	0.4372	-	0.4465	0.4392	0.4348	0.4442
MixHop	0.4317	0.4318	0.4289	-	0.4361	0.4279	0.4377	0.4287
GAT	0.4246	0.4272	0.4262	-	0.4221	0.4276	0.4314	0.4295
MLP	0.5427	0.5429	0.5338	-	0.5357	0.5400	0.5336	0.5414

Table S8. Performance of different GNN models across multiple random seeds on Baron-human4 dataset.

Model	Seed 1	Seed 2	Seed 3	Seed 4-26	Seed 27	Seed 28	Seed 29	Seed 30
GBK-GNN	0.7568	0.7560	0.7545	-	0.7551	0.7568	0.7582	0.7543
H_2GCN	0.6663	0.6765	0.6689	-	0.6592	0.6618	0.6650	0.6588
GraphSAGE	0.4645	0.4641	0.4699	-	0.4680	0.4576	0.4622	0.4590
GCN	0.4587	0.4581	0.4636	-	0.4695	0.4664	0.4680	0.4589
MixHop	0.4507	0.4469	0.4439	-	0.4496	0.4576	0.4514	0.4460
GAT	0.4768	0.4741	0.4762	-	0.4759	0.4765	0.4769	0.4730
MLP	0.5113	0.5129	0.5124	-	0.5124	0.5165	0.5130	0.5129

Table S9. Performance of different GNN models across multiple random seeds on Baron-mouse1 dataset.

Model	Seed 1	Seed 2	Seed 3	Seed 4-26	Seed 27	Seed 28	Seed 29	Seed 30
GBK-GNN	0.6920	0.6999	0.6854	-	0.6929	0.6887	0.6890	0.6865
H_2GCN	0.4895	0.4778	0.4950	-	0.4922	0.4944	0.4854	0.4806
GraphSAGE	0.4487	0.4531	0.4420	-	0.4419	0.4566	0.4627	0.4491
GCN	0.4567	0.4561	0.4583	-	0.4585	0.4594	0.4575	0.4577
MixHop	0.4425	0.4391	0.4395	-	0.4418	0.4451	0.4529	0.4390
GAT	0.4235	0.4272	0.4252	-	0.4243	0.4252	0.4275	0.4184
MLP	0.4863	0.4784	0.4814	-	0.4824	0.4588	0.4750	0.4726

Table S10. Performance of different GNN models across multiple random seeds on Baron-mouse2 dataset.
